# Potential and limitations for monitoring of pesticide biodegradation at trace concentrations in water and soil

**DOI:** 10.1007/s11274-022-03426-x

**Published:** 2022-10-20

**Authors:** Andrea Aldas-Vargas, Baptiste A. J. Poursat, Nora B. Sutton

**Affiliations:** Environmental Technology, Wageningen University & Research, P.O. Box 17, 6700 EV Wageningen, The Netherlands

**Keywords:** Molecular-tools, Molecular-monitoring, Biodegradation, Pesticides, Trace-concentrations

## Abstract

Pesticides application on agricultural fields results in pesticides being released into the environment, reaching soil, surface water and groundwater. Pesticides fate and transformation in the environment depend on environmental conditions as well as physical, chemical and biological degradation processes. Monitoring pesticides biodegradation in the environment is challenging, considering that traditional indicators, such as changes in pesticides concentration or identification of pesticide metabolites, are not suitable for many pesticides in anaerobic environments. Furthermore, those indicators cannot distinguish between biotic and abiotic pesticide degradation processes. For that reason, the use of molecular tools is important to monitor pesticide biodegradation-related genes or microorganisms in the environment. The development of targeted molecular (e.g., qPCR) tools, although laborious, allowed biodegradation monitoring by targeting the presence and expression of known catabolic genes of popular pesticides. Explorative molecular tools (i.e., metagenomics & metatranscriptomics), while requiring extensive data analysis, proved to have potential for screening the biodegradation potential and activity of more than one compound at the time. The application of molecular tools developed in laboratory and validated under controlled environments, face challenges when applied in the field due to the heterogeneity in pesticides distribution as well as natural environmental differences. However, for monitoring pesticides biodegradation in the field, the use of molecular tools combined with metadata is an important tool for understanding fate and transformation of the different pesticides present in the environment.

**Graphical abstract:**

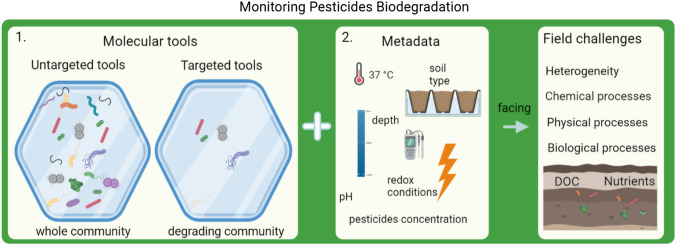

## Introduction

Pesticides consumption is increasing worldwide in order to ensure food production. The rise in the demand for agro-products and changing regional climate has resulted in an increased consumption and application rate of pesticide. For instance, in the Netherlands, the amount of pesticides used in 2019 was almost 9 k tonnes; while Slokavia, which is similar in size and agricultural area, used slightly less than 2 k tonnes (FAO 2021). After application, pesticides can travel in the environment from agricultural soil to surface water and groundwater systems. The fate of the pesticides depends on the environment where they are (Gavrilescu 2005). For instance, pesticides can be adsorbed in matrixes such as soil/sediment or be transported with water. While some pesticides can be transformed, other pesticides or their transformation products can remain in the environment (González-Rodríguez et al. 2011). Persistent pesticides or metabolites threaten the ecosystem as well as the drinking water production from surface water and groundwater sources (Loos et al. 2010; Sjerps et al. 2019).

In the environment, the pesticides’ transformation processes can be physical, chemical or biological (Kumar et al. 2018). Biodegradation is a natural biological process and is one of the most important processes for removing pesticides from the environment (Doolotkeldieva et al. 2021; Saibu et al. 2020). Complete pesticide degradation or transformation processes depend on compound properties as well as environmental biotic and abiotic factors. This process is dependent on contaminant concentration, environmental conditions and microbial community composition and activity (Kowalczyk et al. 2015; Scow and Hicks 2005). Pesticides concentration in the environment is heterogeneously distributed due to their seasonal and geographical application and environmental factors. This heterogeneous distribution is a key factor influencing microbial communities’ exposure to pesticides as well as metabolic potential and activity of these communities. Hence, since environmental conditions and pesticide concentration influence microbial communities composition and overall activity (Verma et al. 2014), biodegradation will differ from one ecosystem to another. For example, agricultural soil microbial communities are considered as more active compared to an oligotrophic environmental such as groundwater aquifers. Therefore, monitoring pesticides biodegradation by native microbial communities in different environments is of importance to better understand pesticides fate and impact on communities under different condition (Fenner et al. 2013).

This review aims to summarize the state-of-the-art on the use of molecular-based assays to monitor bacterial pesticide biodegradation in the field and in laboratory. Our research focuses on bacteria, and not on the influence of other organisms such as fungi or plants on pesticides biodegradation. We define molecular tools, as DNA or RNA based techniques that can or had been used for microbial pesticide biodegradation monitoring. Targeted molecular tools are used for measuring a specific gene or microbial group using known probes while non-targeted explorative molecular tools are high-throughput tools that aim for measuring genes without discrimination or prerequired known probes. Furthermore, despite the valuable contribution of other techniques such as traditional community analysis (T-RLFP, DGGE) (Karczewski et al. 2017), they will not be part of the scope of the present manuscript, due to earlier documentation (Baxter and Cummings 2008; Karczewski et al. 2017; Schütte et al. 2008). To fulfil the main goal of this review, the following questions will be answered, (1) how molecular tools can contribute for monitoring pesticide biodegradation (2) what techniques are available for monitoring pesticide biodegradation (3) what are the advantages and limitations of these tools for use in field monitoring of pesticide biodegradation, and (4) what future opportunities are there to develop new tools for monitoring pesticide biodegradation. This study will not discuss in detail each existing “omics” tools that could be applied for pesticide monitoring, as it is already reviewed (Rodríguez et al. 2020; Vilchez-Vargas et al. 2010). The primary goal of this review is to describe the current potential and limitations of molecular tools for monitoring pesticide degradation and to indicate the future opportunities for developing pesticide biodegradation molecular monitoring tools.

## Relevance of using molecular tools for monitoring pesticide biodegradation

**Fig. 1:**
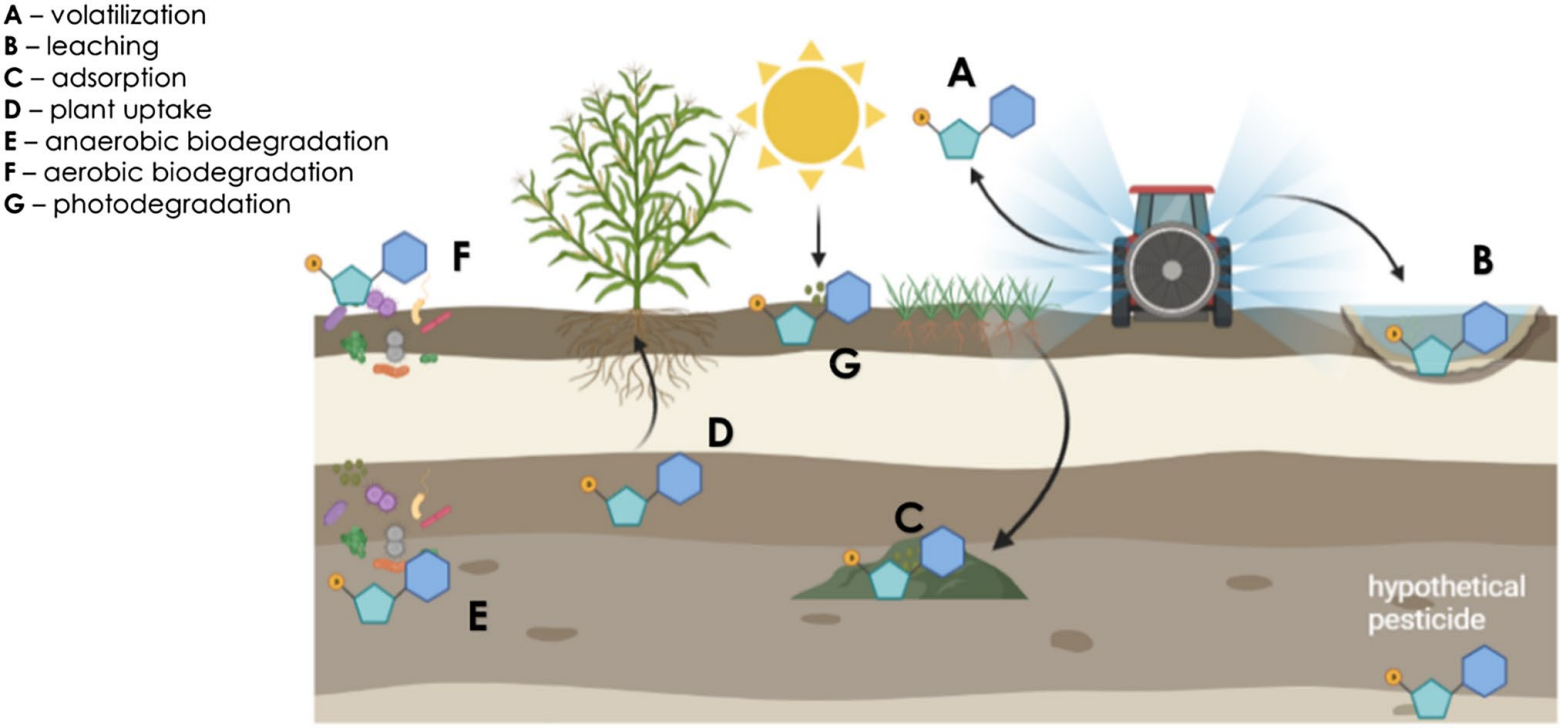
Scheme of the processes influencing the fate of pesticides in the environment after its application. The chemical icon represents a hypothetical pesticide structure. Figure created with Biorender.com

Monitoring of biodegradation of organic contaminants traditionally relied on measuring changes in contaminant concentration and identification of known metabolites. In the case of pesticides, that type of information does not provide evidence that can be directly related to biodegradation alone (Bertelkamp et al. 2016; Scow and Hicks 2005). Traditional chemical monitoring tools alone are insufficient to fully assess pesticide biodegradation in the environment. Pesticides biodegradation cannot be fully assessed by estimating changes in contaminant concentration in the environment (Helbling 2015). Monitoring the changes in pesticides concentration is valuable in case of contaminant point source, and assuming homogeneous distribution in a system. Such monitoring immediately after application and/or days later provides information about pesticides distribution in the system but not much about pesticides biodegradation activity or potential. The reason is that many processes can influence pesticide concentrations in the environment besides biodegradation, such as volatilization, leaching, sorption, and dilution among others (Fig. 1) (Fenner et al. 2013; Wang et al. 2014, 2020). Hence, changes in concentration cannot solely be attributed to microbial biodegradation activity. Furthermore, biodegradation activity of a microbial community can increase after exposure to pesticides (Imfeld and Vuilleumier 2012; Mauffret et al. 2017; Poursat et al. 2019; Tuxen et al. 2002). This means that the biodegradation rate of a pesticide is depending on microbial adaptation and on the enrichment of specific degraders. Thus, measuring pesticides presence or concentration cannot solely assess the development of microbial biodegradation capacity or activity over time. Therefore, complementary molecular tools are needed for monitoring pesticides biodegradation.

Biodegradation of pesticides also cannot be assessed by the sole measuring of secondary metabolites or transformation products in the environment. Metabolite quantification is only possible when the biodegradation pathways are known and without full mineralization of the transformation products (Kolvenbach et al. 2014). Unfortunately, most pesticide degradation pathways are only described for aerobic conditions, which limits insights on anaerobic pesticides biodegradation. Although there are available platforms for non-target compound screening (Helmus et al. 2021), degradation products might be present at trace concentrations, making detection and quantification challenging, even with modern analytical techniques. Finally, degradation processes have been found to vary greatly for the same pesticide between different soil types (Bælum and Jacobsen 2009), emphasizing the diversity of microbial metabolism in heterogeneous systems (Dechesne et al. 2014).

Molecular tools are a powerful asset to monitor pesticide biodegradation in the environment. Biodegradation processes rely on microorganisms and microbial enzymes to transform pesticides (Vandermaesen et al. 2016). Therefore, studying microorganisms’ metabolic capacity and activity can, in combination with chemical data, support our understanding of pesticide biodegradation in heterogeneous environments. Microorganisms can be identified and characterised using cultivation-dependent and cultivation-independent techniques. However, as only a small fraction of microbes can be cultivated under laboratory conditions (Lin et al. 2008; Piel 2011; Sharma et al. 2005), cultivation-independent molecular tools ranging from specific to explorative analyses offer a variety of advantages when assessing pesticide degradation. Besides, recent developments among explorative cultivation-independent techniques have greatly increased the ease with which we can analyse the microbiome (Zhang and Liu 2019). The current review describes state-of the-art DNA and RNA based techniques for pesticides biodegradation monitoring. Techniques that are especially attractive due to recent advances in high-throughput assays and low-cost sequencing.

The use of molecular tools can contribute to a better monitoring of pesticides biodegradation compared to sole traditional analytical monitoring tools. Molecular tools can for instance facilitate distinguishing biotic from abiotic degradation processes when monitoring known key genes from degradation pathways. Moreover, molecular tools can also provide an overview of the microbial metabolic potential or activity from a contaminated site and allow to monitor them over time. Understanding the biodegradation processes can (1) contribute to the protection of water and soil compartments and (2) revolutionize the science of pesticide remediation. For instance, soil and aquatic environments, previously exposed to pesticides would have less chance to be impacted by the application of the same pesticide. Moreover, by understanding the biodegradation processes in different environments, the effect of pesticide remediation technologies can be better assessed.

## State of the art molecular tools used for monitoring pesticide biodegradation

Information generated using molecular tools is an extra line of evidence to monitor pesticide biodegradation next to metadata (i.e., environmental conditions) and direct quantification. Molecular tools can be targeted, focusing on an individual biomarker within a biodegradation pathway, or untargeted, exploring the entire metabolic capacity. Targeted tools have been developed for a number of historically relevant pesticides, meaning that there are” ready to use” targets available, although these are relatively limited to traditionally used pesticides (Table 1). The lack of available gene biomarkers for some pesticides illustrates that developing gene biomarkers databases is challenging and time consuming. However, once available, monitoring is straight forward and very specific. We use two cases to exemplify each of these points: (1) pesticide biodegradation monitoring of a well-studied compound using existing tools, and (2) the route for developing a novel gene biomarker for a new biodegradation pathway. Additionally, a compilation of studies where different genes can be used as biomarkers for monitoring pesticide biodegradation is provided. Since monitoring by targeted molecular tools can be limited by gene biomarkers, the use of non-targeted exploratory tools that can also contribute to monitoring pesticide biodegradation as described later on this manuscript.

**Table 1:** Examples of pesticides biodegradation monitored by targeted molecular tools

Gene	Pesticide	Study area	Redox condition	Process	Nucleic acid	Relevant references
dxmA thmA prmA prmB prmC prmD	1,4-Dioxane	Microcosms	N/A	Degradation	DNA	Deng et al. (2018); Li et al. (2010)
tfdA	2,4-d 2,4-Dichloro phenoxy acetic acid MCPP Mecoprop	Field Columns Microcosms	Aerobic	Mineralization	DNA RNA	Bælum et al. (2008); Batıoğlu-Pazarbaşı et al. (2013); Gazitúa et al. (2010); Gonod et al. (2006); Hayashi et al. (2016); Kumar and Singh (2016); Pinheiro et al. (2015)
atzA atzB atzC atzD atzE atzF trzN trzD	Atrazine	Field Microcosms	Aerobic Anaerobic	Degradation Mineralization	DNA RNA	(Cheyns et al. 2012); Clausen et al. (2002); Devers et al. (2004); Douglass et al. (2016); Dutta and Singh (2015); Monard et al. (2013, 2010); Sagarkar et al. (2014); Sherchan and Bachoon (2011)
bbdA	BAM	Field Microcosms	Aerobic Anaerobic	Degradation Mineralization	DNA	Ekelund et al. (2015); Ellegaard-Jensen et al. (2017); Horemans et al. (2017); Sekhar et al. (2016); T’Syen et al. (2015)
cahA cehA	Carbaryl Oxamyl Propoxur	Laboratory	Aerobic	Degradation	DNA	Hashimoto et al. (2006); Kim et al. (2017); Rousidou et al. (2016)
mhel	Carbendazim	Laboratory Microcosms	Aerobic	Degradation	DNA	Lei et al. (2017); Pandey et al. (2010); Zhang et al. (2013)
mcd	Carbofuran	Laboratory Microcosms	Aerobic	Degradation	DNA	Derk et al. (2003); Topp et al. (1993)
chd	Chlorothalonil	Laboratory	Aerobic Anaerobic	Degradation	DNA	Fang et al. (2018); Wang et al. (2010)
mse1	Cypermethrin	Laboratory Microcosms	Aerobic	Degradation	DNA	Diegelmann et al. (2015)
ese esd	Endosulfan Endosulfate	Laboratory Microcosms	N/A	Degradation	DNA	Sutherland et al. (2002); Weir et al. (2006)
glpA glpB	Glyphosphate	Laboratory Microcosms	Aerobic	Degradation	DNA	Penaloza-Vazquez et al. (1995)
linA linA2 linB	HCH Hexachlocyclo-hexane	Field Microcosms	Aerobic Anaerobic	Degradation	DNA	Gupta et al. (2013); Mertens et al. (2006); Sangwan et al. (2012)
pdmA pdmB ddhA adoQ adoT adoA1 adoA2 adoB adoC adoE tdnC tdnE tdnE tdnF tdnL	Phenyl urea herbicides Isoproturon Linuron	Field Microcosms	Aerobic	Degradation Mineralization	DNA	Bers et al. (2012); Fujii et al. (1997); Fukumori and Saint (1997); Gu et al. (2013); Khurana et al. (2009); Pesce et al. (2013)
hylA dcaQ dcaT dcaA dcaB dcaR ccdC ccdD ccdF ccdR	Prosulfocarb Pendimethalin	Field Microcosms	Aerobic	Mineralization	DNA	Fang et al. (2018); Nour et al. (2017)
rdpA sdpA	MCPP Mecoprop	Field Microcosms	Aerobic Anaerobic	Degradation Mineralization	DNA RNA	Paulin et al. (2011, 2010)
hdl-I hdl-II dan dcl cbe prc hdx dhn mfe	Metamitron	Laboratory	Aerobic	Degradation	DNA	Fang et al. (2018, 2016)
mdeA mdeB mdeC mdeD	MDE 3-methyl-diphenulether	Laboratory	Aerobic	Mineralization	DNA	Yang et al. (2016)
trzA	Melamine	Field	Aerobic	Degradation	DNA	Dodge et al. (2012)
mpd mph ophC2	Methyl parathion	Laboratory Microcosms	Aerobic	Degradation	DNA	Shen et al. (2010); Wei et al. (2009); Zhongli et al. (2001)
cpd opd opdA opdB opdE hocA opaA opaB dmhA hocA imh pdeA pepA pehA adpB phnA-Q	Organo-phosphorus pesticides	Microcosms	Aerobic	Degradation	DNA	Acharya et al. (2014); Chen et al. (1990); Cheng et al. (1993); Chino-Flores et al. (2012); Dotson et al. (1996); Fang et al. (2018); Jao et al. (2004); Kwak et al. (2012); Mulbry (1992); Parker et al. (1999); Tehara and Keasling (2003)
pcd	Phenmedipham	Laboratory	Aerobic	Degradation	DNA	Pohlenz et al. (1992)
atzB1	Simazine	Microcosms	N/A	Degradation Mineralization	DNA	Martínez-Iñigo et al. (2010)
suaC subC sulE	Sulfonylurea herbicides	Laboratory	Aerobic	Degradation	DNA	Hang et al. (2012); Omer et al. (1990)
thcB thcB thcD	Thiocarbamates	Laboratory	N/A	Degradation	DNA	Shao and Behki (1996)

### Monitoring atrazine biodegradation

Atrazine is one of the most studied pesticides and is used as herbicide in perennial crops such as maize, sorghum and sugar cane (Singh et al. 2018). Due to the diversity of the crops where it is used, it has been applied worldwide. In 1950 it was commercialized for the first time and since 2003 it was banned from the European Union due to its toxicity (Sass and Colangelo 2006). However, years after its prohibition it can still be found in contaminated agricultural soils. Also, due to rainfall and percolation, this compound has been transported to groundwater reservoirs threatening drinking water production (Loos et al. 2010; Sjerps et al. 2019). The fact that atrazine is mostly used in perennial crops, results in regular applications and consequently in an increased exposure for soil microbial communities, which could lead to development of degradation capacity. In literature, it has been reported that soils treated with atrazine rapidly mineralised this herbicide, compared to non-treated soil (Piutti et al. 2003; Topp et al. 2000).

Targeted molecular tools are widely used to study atrazine biodegradation in laboratory and field studies. Several genes involved in the aerobic degradation pathway of atrazine have already been identified in the *Pseudomonas* sp. ADP strain and was then used as gene biomarkers as atrazine-degrading genes are highly conserved in diverse genera of bacteria (de Souza et al. 1998; Ma et al. 2017). The biomarker genes *atzA* to *atzF*, cover different steps of the atrazine biodegradation pathway through to complete mineralization (Devers et al. 2004). Next to *atz* genes, the genes *trzD* and *trzN* have also been reported to play a role in atrazine biodegradation in some bacterial strains, either combined with *atz* genes or without them (Table 1) (Piutti et al. 2003; Sajjaphan et al. 2004). The expression of the biomarker genes *atzABCDEF*, which are located on a self-transmissible plasmid, has been tested on different bacterial strains isolated from soil and showed catabolic activity immediately after the addition of atrazine (Devers et al. 2004).

Targeted analyses of the above mentioned gene biomarkers can be used to monitor specific atrazine biodegradation pathways using qPCR and RT-qPCR (Devers et al. 2004; Monard et al. 2013). By quantifying known genes, associations can be drawn between the increase or decrease in presence (qPCR) or expression (RT-qPCR) of degrading genes and environmental data (i.e., pesticides concentrations, redox conditions among others). Atrazine biodegradation can be monitored for instance by using the identified biomarker genes (i.e., *atzABCDEF, trzD trzN*). However, we cannot ignore the possibility that other biodegradation pathways exist with yet unidentified degradation genes. Moreover, qPCR, while highly specific and quantitative, only allows the testing of one known target per assay, leading to slow data generation.

High throughput explorative molecular tools can be used to quantify the expression of more than one gene per assay while also being specific. Tools such as the GeoChip (functional micro-array) can incorporate atrazine degrading biomarker genes among its targets. The use of a functional-microarray allows for a broader screening of the microbial metabolism while still monitoring specific atrazine degradation activity (Lu et al. 2012). Functional micro-arrays are able to monitor several targets simultaneously with high technical reproducibility (Tu et al. 2014). The design of a specialized array able to assess pesticides biodegradation, although feasible, is costly due to the need of technological advances and computational power to create a sensitive and compound-specific probe (Dugat-Bony et al. 2012; Van Nostrand et al. 2012). Despite those challenges, complementary use of explorative molecular tools could contribute for monitoring a large set of known genes or microbial communities with potential biodegradation activity.

Targeted and explorative molecular tools contribute to the monitoring of pesticide biodegradation and therefore help on the protection of water and soil compartments. A combination of both tools would facilitate not only monitoring well-studied pesticides like atrazine but also other more emergent pollutants. Moreover, for well-known pesticides complementary information can be obtained in terms of what other microbial processes might also be occurring and what is the effect of those processes in pesticide biodegradation. Furthermore, molecular tools support a better assessment of pesticide bioremediation technologies, such as natural attenuation, biostimulation and bioaugmentation in soil microcosms. Biostimulation as a remediation technology was used to enhance atrazine biodegration by the addition of molasses (Nousiainen et al. 2015). When using monitoring tools to evaluate biostimulation, it was observed that atrazine degradation gene copy numbers did not increase as expected (Nousiainen et al. 2015). The activity of the monitored genes however remained unknown since DNA was used instead of RNA. This exemplifies the limitations of quantifying the amount of target genes instead of the amount of target enzymes. Other laboratory studies conducted bioaugmentation with an atrazine-degrading consortium (i.e., *Arthrobacter* sp. AK_YN10, *Pseudomonas* sp. AK_AAN5 and *Pseudomonas* sp. AK_CAN1) together with biomarker genes. Bioaugmentation resulted in 90% of atrazine degradation in pre-exposed soil mesocosms, where the marker genes could be quantified along the experiment (Sagarkar et al. 2014). Such laboratory experiments show the use of molecular monitoring tools to better assess the effect of bioremediation technologies in the laboratory and suggests its potential for field application.

### Discovering a novel BAM biodegradation pathway

The first central step to pesticide biodegradation monitoring with targeted tools is to discover biodegradation pathways. To measure the biodegradation of a specific pesticide, several non-trivial discovery steps are required, including gene decoding and heterologous expression, before using it as a biomarker. In many cases it is necessary to cultivate microorganisms, limiting thus the study to culturable bacteria only. Recent work has successfully designed new biomarker targets for BAM (2,6-dichlorobenzamide), which is a recalcitrant degradation product of the pesticide dichlobenil commonly found in the environment at concentrations ranging from ng to µg/l (Sjerps et al. 2019).

To identify the BAM biodegradation pathway in soil, an extensive culturing experiment was performed over several years and published in numerous articles. We will provide here a short summary of the different experiment required for the identification of BAM biodegradation pathway in soil. The first task was to decode potential degraders. For this, a BAM mineralizing culture, derived from dichlobenil contaminated soil samples, was enriched in laboratory. Afterwards, the identified BAM degrader strain, *Aminobacter* sp. MSH1 (Sørensen et al. 2007)*,* was selected for further exploration of the BAM amidase biomarker gene *bbdA* and enzymes associated to the BAM biodegradation-mineralization pathway (T’Syen et al. 2015). The degrader gene was amplified and cloned to verify its role in the biotransformation of BAM to 2,6-dichlorobenzoic acid (T’Syen et al. 2018, 2015). The degradation steps leading to complete BAM mineralization remained unknown until a recent publication from Raes et al. (2019). This extensive experiment, only summarized here, allowed for the identification and characterization of one gene-enzyme couple responsible for one catabolic reaction in soil. Identification and isolation of one catabolic gene required the use of several tools such as next generation sequencing (NGS), qPCR as well as the use of tandem mass spectrometry (MS–MS) for a full proteomic and metabolomic analysis. The use of molecular tools was key to monitor BAM biodegradation in sand filter columns similar to the ones from drinking water facilities (Ellegaard-Jensen et al. 2020). This study is only one example on how monitoring molecular tools can facilitate the use of pesticides bioremediation technologies and contribute to water protection.

In conclusion, as shown in this example, discovering new biodegradation pathways is not an easy task. In the case of BAM, the isolation and characterization of a specific natural degrading bacteria to the development of a full biodegradation pathway for this strain took around twelve years and several extensive cultivation-dependant experiments that used diverse genomic, proteomic, metabolomic and analytical chemistry tools. Although maybe this biodegradation pathway might differ under different environmental conditions, it is now possible to follow the expression of BAM catabolic biomarker genes or associated microorganisms in laboratory experiments (Ellegaard-Jensen et al. 2020) and in the environment (Sjøholm et al. 2010).

Table 1 provides other example of catabolic genes that were identified by the scientific community. This list represents potential targets validated for molecular monitoring of the biodegradation of pesticides. Considering that finding catabolic genes takes many years as exemplified with BAM, these genes should be further used to monitor bioremediation technologies. In Table 1, many genes had been tested in laboratory but not yet in microcosm experiments like in the atrazine case. Moreover, very few of the listed genes, had been used to monitor biodegradation in field conditions. Challenges on this matter will be further elaborated later in this manuscript. To finalize, we observed that the number of discovered catabolic genes increases every year. However, it is crucial to use microcosm experiments as well as alternative non cultivation-dependant techniques to further explore biodegradation capacity and activity of the microbiome.

### Non-targeted molecular tools for monitoring pesticides biodegradation

The interest in non-targeted, or explorative, molecular tools, such as metagenomics and metatranscriptomics, have increased since the development of next generation sequencing (NGS). These tools have the potential to screen the entire microbial population composition or their metabolic capacity at once without requiring a priori knowledge about the studied community (Zhou et al. 2015). In the field, where not one but several pesticides are present, this feature is very useful to obtain a snapshot of the microbial biodegradation capacity and activity. These tools provide new opportunities and insight to explore the prevalence and distribution of known biodegradation genes for pesticides in complex environments (Fang et al. 2018).

Non-targeted tools are a way to investigate the genetic potential and activity of the microbiome. High throughput explorative tools can help assessing biodegradation as well as other processes that can occur due to contaminant presence. For instance, known genes associated with atrazine biodegradation, *atzABCDEF*, were detected in agricultural soil (Malla et al. 2022) and in the rhizosphere of trees (Aguiar et al. 2020) using metagenomic. Analysis of freshwater and marine sediments was able to detect differences in genes associated with pesticide biodegradation pathways depending on the environmental conditions (Fang et al. 2014). In a different study, research revealed correlations among bacterial communities and the associated pesticide biodegradation genes to the sampling season as well as wastewater characteristics (Fang et al. 2018). In this specific study, 20 samples of activated sludge from various WWTP were collected to perform a metagenomic sequencing analysis and investigate the seasonal dynamics of bacterial communities and their associated pesticides biodegradation genes. Information about genes involved in the catabolic degradation of 10 pesticides, including metamitron, atrazine, isoproturon, linuron, nicosulfuron, organophosphates, pyrethroid, 2,4-dichlorophenoxyacetic acid (2,4-d), carbendazim, and chlorothalonil were collected from NCBI and grouped into a nonredundant protein database. Bacterial 16 s rRNA gene amplicon sequencing reported the presence of bacterial genera known to degrade organic pesticides, according to the NCBI database, with geographical and seasonal variation. A total of 68 subtypes of pesticide biodegradation genes were detected in the activated sludge samples. The most abundant subtype of biodegradation gene was the *dhn* gene, which encodes for dehydrogenase and may be used in the biodegradation of metamitron. Furthermore, in terms of abundance, genes involved in metamitron biodegradation (see Table 1) were the most abundant, followed by the genes related to the biodegradation of linuron. In this way, explorative tools showed to be powerful when studying molecular changes related to pesticide presence and biodegradation. Unfortunately, metagenomic data is still under-utilized due to the lack of conceptual strategies for linking the metagenomic data to study functional traits (Fierer et al. 2014).

Metatranscriptomic is a molecular tool that provide insights on the microbial communities’ structure and their activity. In agricultural soil having a long history of usage of pesticides, cypermethrin I, II, III, IV was identified as one of the main pesticides present (Sharma and Sharma 2018). The agricultural soil was enriched with bacterial genera with potential for the degradation of cypermethrin and also transcripts related to the degradation of aromatic compounds via benzoate were found in high abundance (Sharma and Sharma 2018). In another study where there was point source pesticide contamination, metagenomic and metatranscriptomic data demonstrated that with high pesticide concentration there was also an increase in genes related to degradation of aromatic compounds such as peroxidases, monooxygenases, and cytochrome P450, among others (Russell et al. 2021). Both studies confirm the potential of high-throughput explorative tools to evaluate pesticides degradation, especially when it is known that pesticides are present due to historical pollution or due to point source concentration.

Metagenomics is sometimes referred to as one of the best approaches to discover novel biodegradation pathways. However, the exploration of catabolic pathways by metagenomic analysis is limited by the availability of references in the databases (Fang et al. 2014). One option to overcome this challenge can be to conduct metagenomics sequencing in laboratory experiments where degradation activity can be confirmed by measuring changes in pesticides concentrations for instance. With the adequate use of controls, potential genes associated with degradation of pesticides can be identified in the experiments where biodegradation activity is detected. Moreover, if metatranscriptomics are used, the microbial catabolic activity can be assessed by measuring the active gene pool the community. One of the advantages of using high-throughput explorative tools is that a system could be studied as a whole, creating a snapshot of a community and individual microbial processes. Furthermore, metagenomics could be used to predict degradation rate of specific compounds (Jeffries et al. 2018). Metagenomic sequencing price is rapidly decreasing, showing the potential to monitor all biodegradation capacity in a sample without having to preselect the targeted genes (Fang et al. 2018). Promoting the use of such tools in controlled laboratory experiments could lead to the validation of new candidate genes or enzymes involved in biodegradation processes that can afterwards be used for the monitoring of pesticides biodegradation.

A main challenge in the application of non-targeted molecular tools, is that finding associations between microbiomes and biodegradation processes can be extremely challenging. First, assuming that there seems to be an association between a gene and a transformation product, this phenomena can be responding to microbial ecology dynamics rather than specific microbial degradation processes (Johnson et al. 2015). Also the interpretation of the massive amounts of data generated is not an easy task, especially considering that this can lead to an infinite possibilities of post hoc analysis (Bell et al. 2015). In that sense, the generated data can become useless if there is no chemical and biological metadata associated with the produced molecular data. Despite its current limitations, metagenomic is still one of the most promising approaches for uncovering candidate genes or gene products related to pesticide biodegradation.

To use non-targeted tools in a more efficient way, genes databases created by measuring the prevalence of known biodegradation genes and their associated microorganisms are necessary for the construction of biodegradation pathways (Fang et al. 2014). Currently, next to theoretical pesticide biodegradation models, there is also the possibility of building models that could incorporate the abundance and expression of functional degradation genes for a better understanding on pesticides biodegradation (Chavez Rodriguez et al. 2020). Constructed biodegradation genes databases, as developed in Box 1, and the use of models will help the scientific community to follow the catabolic activity and potential of communities. At the same time, using laboratory data to model biodegradation shows that a combination of data science and molecular tools can facilitate monitoring pesticides biodegradation. For that reason, the in-silico tools will be further discussed in Box 1.

### Limitations on the application of monitoring pesticide biodegradation using molecular tools

We have shown that a variety of molecular tools are available to monitor pesticide biodegradation, such as atrazine or BAM (Table 1). However, successfully applying these tools to monitor biodegradation in the field is a complex task. One of the first challenges stems out of the way molecular tools are developed. Generally, microbiome monitoring tools are developed and optimized using liquid isolates of pure cultures, which inherently involves extra time and effort to validate methods for other sample types (Fenner et al. 2013), such as soil and sediment. This means, that prior to sampling, several aspects, that are not always related to pesticides biodegradation, need to be considered and arranged to make a sampling campaign successful. For certain tools, such as qPCR, guidelines had been proposed to obtain better experimental practice and more reliable result interpretation (Bustin et al. 2009). However, it should be mentioned that targeted tools can also overestimate the pesticide degradation capacity of a community, because DNA quantification does not discriminate between the active microbial population and the dormant or even the dead one (Pietramellara et al. 2009). In the case of RT-qPCR, the instability and short life span of RNA molecules, which is quickly degraded by RNAses, results in under estimation of microbial degradation activity. Thus, these considerations need to be considered before drawing conclusions based on experimental data.

**Fig. 2:**
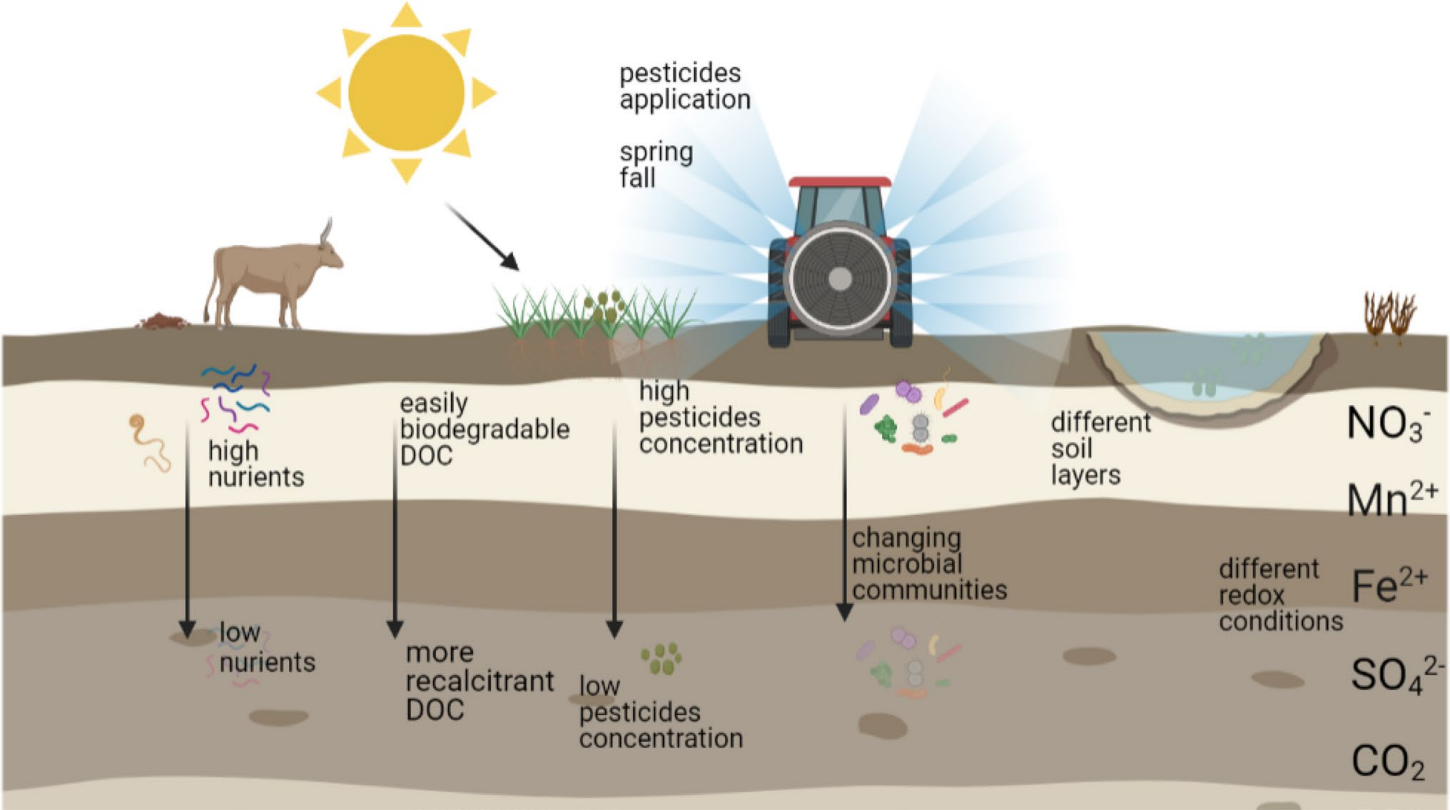
Scheme representing the field heterogeneity in terms of environmental conditions and pesticides distribution. Figure created with Biorender.com

An additional well-known challenge in microbial ecology is the difficulty to accurately sample a representative microbial community from the field. Heterogeneous environmental conditions in the field result in microbial communities that differ within very small scales (Fig. 2). Thus, the microbial communities biodegrading pesticides in the environment are not homogeneous (Dechesne et al. 2014), due to local variation in environmental conditions (Vandermaesen et al. 2016) which can influence the spatial and temporal distribution of degrading cells and genes (Fierer 2017). Vertical variations in soil results in a constituent decline of pesticide biodegradation activity with increase of soil depth (Dechesne et al. 2014). Soil and sediment spatial distribution of pesticide degrading genes has been shown to diverge at the centimetre scale (Batıoğlu-Pazarbaşı et al. 2012; Sjøholm et al. 2010). Soil top layer communities have more chance to be exposed to pesticides after application, which results in development of biodegradation activity in the top layer whereas samples taken just below the surface would not show degradation capacity (Imfeld and Vuilleumier 2012; Mauffret et al. 2017; Tuxen et al. 2002). Differences in microbiome’s overall and biodegradation activity were observed with groundwater depth, due to changing redox, the decrease in carbon and pesticide concentration (Aldas-Vargas et al. 2022). Furthermore, the degrading population seems to be more affected by the decrease in carbon concentration than the rest of the heterotrophic population (Dechesne et al. 2014), as has been observed for the degradation of a phenoxy acid herbicide MCPA (2-methyl-4-chlorophenoxyacetic acid). This means that multiple samples from different depths must be analysed to truly assess biodegradation at a given location (Fig. 2). Horizontal variation in biodegradation activity are more commonly associated with variation in pesticide application, sampling design and sampling site, and not with environmental gradient as it is the case for vertical variations (Dechesne et al. 2014). For example, high variation of 2,4-d concentration in the horizontal plane is a sign of heterogeneity of biodegradation activity and capacity across the local microbial community (Dechesne et al. 2014). To tackle these challenges, an increased number of sample location and time points is recommended.

Finally, a last challenge is that in field conditions with environmentally relevant pesticide concentrations, only a small portion of the community may biodegrade pesticides. Thus, the abundance of the degrading population within a community might be below the detection limit of PCR based tools, as it was showed for *tfdA* genes associated with phenoxy acid degradation (Batıoğlu-Pazarbaşı et al. 2012). Hence, pesticide biodegradation may remain overlooked due to experimental detection limits. This challenge can be addressed by enriching the low abundance degrading population in laboratory or extracting and concentrating high volumes of field samples. However, progress in the field of molecular tools might lead to the development of novel optimized amplification techniques able to detect and quantify low abundance populations.

### Future perspectives for the use of molecular tools for monitoring pesticides biodegradation

In the previous sections we presented examples of some pesticides for which, after years of work, gene biomarkers have been identified that can be used for monitoring biodegradation. Fortunately, the list of pesticides from which mainly aerobic biodegradation can be monitored (Table 1) is longer than the examples presented above (i.e., atrazine and BAM). However, finding targets for emerging contaminants, requires constant work to keep targeted monitoring feasible, as thoroughly discussed previously in this manuscript.

Pesticides can travel through aerobic and anaerobic environments (Fig. 1). For that reason, research needs to shift towards elucidating anaerobic pesticide biodegradation pathways. The gene biomarkers summarized in Table 1 show a clear trend about research in aerobic degradation, which limits those biomarkers’ application to monitoring aerobic environments. Taking atrazine as an example again, it was demonstrated that it can be biodegraded under nitrate reducing, sulphate reducing and methanogenic conditions (Boopathy 2017). However, biodegradation was mainly monitored by changes in concentration. As the study was conducted under controlled laboratory experiments and using a specific inoculum, conditions are not comparable with the situation in the field. Thus, although it’s been acknowledged that atrazine can be biodegraded in anaerobic conditions, anaerobic biodegradation cannot be monitored in the environment because there are no anaerobic gene biomarkers available.

Molecular targets allow for the monitoring of biodegradation processes. Currently, the entire atrazine biodegradation pathway could be monitored for aerobic environments since the genes and metabolites have been described for the entire degradation pathway. This means that atrazine biodegradation could be potentially followed step by step by monitoring known genes (Table 1). This is again, the ideal example, which unfortunately is not applicable for other pesticides. We observe in Table 1, that for many pesticides there is only one biomarker gene that can be monitored, as seen in the BAM example. Unfortunately, many of the studies focus on one or a few degradation steps (Table 1), while understanding the biodegradation pathway to complete pesticide mineralization remains unknown. Moreover, in some cases, pesticide biodegradation can be conducted by either different genes that encode enzymes with a similar function or by yet non-described enzymes for alternative degradation pathways (Benner et al. 2015; Hedegaard et al. 2018), which makes monitoring relying in specific targets difficult.

In the atrazine example, it was mentioned that not only the biomarker presence was detected, but also the activity was measured based on RNA (Nousiainen et al. 2015; Sagarkar et al. 2014). For that, qPCR as well as RT-qPCR were used as monitoring tools. In Table 1, it is observed that monitoring often focuses on DNA; thus gene presence is monitored but activity is not. Using RNA as a starting material would allow the monitoring of actual biodegradation activity, instead of biodegradation potential using DNA. A challenge of using RNA is that the practical laboratory requirements of working fast due to the instability of RNA (Tan and Yiap 2009) and maybe additional concentration steps or a bigger sample due to the low concentrations of RNA in environmental systems (Griebler and Lueders 2009). Thus, sampling techniques need to be optimized, especially in cases where low biomass is present, to increase DNA concentration and quality, and unlock the possibility to use RNA-based molecular tools as well.

In this review the state-of-the art of monitoring pesticide biodegradation was presented. Some of the success stories as well as the limitations regarding the use of molecular tools for monitoring pesticide biodegradation were also addressed. We hope that future scientific advances will see the current limitations as research opportunities to develop new molecular tools, and that these tools take a more predominant role in understanding pesticide biodegradation processes in the environment. We would recommend looking at the chemical structure of the compound of interest to compare it with the list of pesticides for which there are markers available. In the case of mecoprop-p (MCPP), for instance, *tfdA* genes associated with 2,4-d, which is also a phenoxyalcanoic acid herbicide, were used for monitoring MCPP degradation (Rodríguez-Cruz et al. 2010). Although this approach may not be straight forward, it can be useful to gain initial insights about the general pesticides biodegradation capacity of a system.

Finally, due to the complexity of biodegradation processes, monitoring needs to be addressed in an holistic way, meaning combining molecular tools with state-of-the-art analytical chemical tools as well as high-tech data analysis facilities (Fenner et al. 2021). In that sense, the use of chemical analyses such as compound specific isotope analysis (CSIA) and high-resolution mass spectrometry (MS) generate metadata that is necessary for confirming biodegradation. To this end, we expect a joint effort from the scientific community studying biodegradation by sharing data from field and laboratory studies together with the associated metadata. In this way, meta-analyses could be conducted aiming to understand which environmental conditions are theoretically favourable for pesticides biodegradation. Moreover, the role of data scientists for curating biodegradation databases (Box 1) and facilitating the incorporation of experimental data into modelling is necessary as well to accelerating advances in studying pesticides biodegradation.

Box 1: In-silico tools for biodegradation prediction Quantification of specific catabolic-gene expression can be correlated with the biodegradation activity of a soil community (Monard et al. 2013). Molecular data have been used to develop model that could predict the biodegradation activity of a community based on its gene expression. However, for now, these models are not fully ready yet and are still exhibiting an erratic relationship between pesticide biodegradation rate, such as 2,4-d and MCPA, and gene expression (Chavez Rodriguez et al. 2020). This increases the difficulty to solely use gene expression quantification as a soil biodegradation assessment and shows the importance of developing further in silico monitoring of biodegradation processes to explore catabolic potential

Due to the increasing number of novel pesticides, traditional, experimental approaches to discover new biodegradation pathways and bioremediation potential are limited because they are time and money consuming and technically challenging. Under the REACH regulation (Registration, Evaluation, Authorisation and Restriction of Chemicals) in silico methods, such as quantitative structure–biodegradation relationships (QSBRs) models, may be used as a substitute for experimental data, and/or as a supplement to experimental data (European Chemicals Agency 2017). For instance, pesticides biodegradability in aquatic environment can be predicted using empirical models, such as Biowin, VEGA and CATALOGIC (Dimitrov et al. 2011; Pizzo et al. 2013). However, there is currently a need to develop new in-silico tools to identify enzymes and microorganisms involved in the biodegradation and biotransformation of those novel chemicals

In silico approaches, such as the Envipath tool (the Environmental Contaminant Biotransformation Pathway Resource; https://envipath.org/), can be used to provide information on specific enzyme-catalyzed reactions involved in the biodegradation of organic contaminants (Wicker et al. 2016). The enviPath tool, that is developed by EAWAG (the Swiss Federal Institute of Aquatic Science and Technology) and was recently merged with the Biocatalysis/Biodegradation Database (UM-BBD) from the University of Minnesota (USA) (Ellis et al. 2003; Wicker et al. 2016), provides information about 219 pathways, 1503 reactions, 1396 compounds, 993 enzymes and 543 microorganisms and can as well be used to predict unknown biotransformation from a specific organic pollutants (Gao et al. 2010)

Moreover, computational analysis could also be used to give a potential explanation for the lack of biodegradability of known persistent compounds (Aukema et al. 2017). Some in silico approaches are focusing on the identification of unknown transformation products that might be produced during biodegradation of an organic chemical. However, they are not designed to assess the biodegradation capacity of a specific community in the environment, even if in-silico approach could be used to construct artificial consortia that could bioremediate organic pollutants (Awasthi et al. 2018). Hence, the next challenge is to translate in-silico results to the field and to use such data to predict the bioremediation potential of a community and/or environment. However, in its current state, this approach could help to guide scientist on which analysis to use to gather informative data regarding pesticide environmental fate and behavior. By predicting what specific enzyme-reaction is involved in the biodegradation of a compound of interest, known and unknown genes connected to this function could be traced back in the community through a meta-omic approach. Identification of the organism expressing this gene is a step forward to monitor pesticide biodegradation

## Conclusion

In this review we assessed the state-of-the-art of molecular tools for monitoring pesticides biodegradation in the field. Investigating environmental biodegradation pathway cannot solely be achieved by analytical chemistry and biogeochemistry. Investigating the microbiome is not only crucial to monitor the biodegradation capacity and activity of indigenous microbial communities, it also supports discovering new biodegradation pathways. Cultivation-based tools and laboratory-based discovery of biodegradation pathways had been valuable and will probably continue to be used in the future, ideally combined with multi-omics analyses. Even if there is still progress to be made, detecting, measuring and predicting biodegradation of pesticides in the field will improve our understanding of the fate and transformation of these pollutants, which is crucial in protecting and remediating polluted environments. Monitoring pesticide biodegradation will as well help for the design of optimized environmental technologies towards the treatment of pesticides in soil and water. Microbiome used to be a black box mostly inaccessible to environmental technologists. Using molecular tools, studies can be conducted for monitoring biodegradation capacity and activity, turning the black box into a grey one.
